# Psychiatry in India: Need to focus on geriatric psychiatry

**DOI:** 10.4103/0019-5545.31612

**Published:** 2006

**Authors:** Asit Baran Ghosh

**Affiliations:** President IPS, Rohini Complex, Flat no. C-2/5A. Kolkata 700014, West Bengal e-mail: drasitghosh@rediffmail.com

Respected Chairperson and Members of the Indian Psychiatric Society (IPS), it will perhaps be an act of audacity on my part to speak to the gracious and distinguished gathering assembled here at the 58th Annual Conference. But I cannot control the temptation to express my thoughts and feelings. I esteem it a rare privilege to address this audience of erudite, diligent and dignified persons.

On this occasion, I convey my deepest gratitude and respect to my revered teachers without whose help and guidance it would not have been possible for me to stand in front of you. But, I feel I am still a pigmy, nowhere near the depth of their knowledge and wisdom. So please bear with me, as I share with you my observations.

The IPS, the leading national platform for mental health professionals, has a colourful heritage of 57 years during which many eminent psychiatrists have adorned the presidential leadership of this national body. I consider myself insignificant in comparison to those stalwarts but, at the same time, feel a deep sense of obligation to all of them whose support and encouragement has helped me to shoulder this heavy responsibility as the President of the IPS. I honestly believe that all-round cooperation from our Office bearers and all Members will enable me to hold high the mission of the IPS. I also thank the IPS for its global recognition and importance in the world psychiatry scenario.

We are passing through a period of globalization which has a strong influence on the healthcare delivery system at every level. Though the IPS is concerned mainly with the advancement of scientific knowledge and professional solidarity of mental health specialists, I think the time has come when we have to be more concerned about the interest of the community. Today, I will try to share with you my thoughts in this context and hope the IPS will pay avid attention to these issues.

## ROLE OF THE IPS

Mental health is not a priority agenda everywhere. We know that a mental health policy exists in 59.5% of the WHO countries covering about 85% of the world population.^1^ In India, we still lack a comprehensive mental health policy both at the central and state level. We need a definite and optimum central mental health policy with clear-cut directives for treatment coverage, rehabilitation and mental health promotion and advocacy with a focus on the rural population.

The IPS has a renowned work record in this connection, namely the negotiation of a mental health policy with the Central Government. The IPS Parliamentary Committee can take up this issue with the Central Government for effective planning, policy-making and implementation. The National Mental Health Programme should be strengthened and cover more states. A similar need is felt for a Central Substance Abuse Policy and Therapeutic Drug Policy. The IPS taskforce on clinical guidelines can negotiate for a Central Therapeutic Drug Policy for the entire country. Both the Central and State Governments should be urged to make the essential list of psychotropic drugs available at the primary care level as well.

I am convinced that though the IPS, through its different subcommittees, takes a lot of initiative for mental health education and training of professional colleagues, it is also true that our activities are restricted mainly to urban centres and there exists a considerable gap between the community and us.

We should organize our activities in such a way that our mental health promotion and advocacy programmes involve more and more segments of the community. An important issue in this context is mental health training for primary care physicians.The IPS can take a leadership role in negotiating this with State Governments. We should not forget that 80% of our population is in the rural areas.

The role, activities and strength of the IPS in the national context are highly encouraging. We have branches in almost all the states.

In view of the enormous number of patients with mental illness and the paucity of different categories of mental health professionals,^2^ we have a very critical, unbalanced mental health delivery system in this country. A cohesive effort from the government, non-governmental agencies and the IPS may address some of these issues effectively.

Though the IPS has different subsections with dedicated responsibilities, this is the time when the IPS should think of a specialized college (such as the college of GPs in the IMA) which will aim, with the help of the government, to train a mental health workforce in clinical psychology, psychiatric social work, etc.

Capacity building with different NGOs working in mental health and the private sector will also be beneficial. A special task force may be formed for feasible studies in this context.

I would like to draw your kind attention to the following issues, the implementation of which will help in consolidating the aim of the IPS of preventing mental illness and promoting mental health.

1. The Mental Health Act (MHA) both at the Central and State levels has been in force in India during the past 10 years. There should be a clear provision for IPS members/fellows to be members of those formulating the MHA, as representatives from the national professional body.
2. *The Indian Journal of Psychiatry* is a prestigious publication of the IPS which is approaching 50 years of service to mental health. This highly academic journal publishes clinical and research material. Common people have no access to this professional literature. I think the IPS should publish a news bulletin for the general public which address issues such as the fight against stigma, discrimination and public education for mental health promotion. State branches may translate public health messages into the vernacular languages for the interest of the local people.
3. Psychiatric education is the cornerstone for generating good mental health professionals. Unfortunately, psychiatry at the undergraduate level is a highly neglected area which needs immediate attention. A few attempts were made in the past by the IPS, but without any sustainable results. We can take it up as a consolidated effort with the Ministry of Health, Medical Council of India (MCI) and University Grants Commission (UGC) for an optimum undergraduate curriculum in psychiatry.
4. In the National Mental Health Programme there is a provision for training medical officers and paramedical staff of PHCs. This can only be a short-term solution. For the long-term delivery of mental healthcare to the rural people, doctors must learn basic psychiatry as part of their undergraduate curriculum. Psychiatry as a subject should be included in the undergraduate curriculum, not only as an optional, but also as an examination subject.
5. Presently, there is no mechanism for quality control in postgraduate studies, nor is there a system for quality assurance of psychiatric education in the country. In view of the recent trend of evidence-based medicine, the entire perspective of teaching is undergoing a radical change and psychiatric education should accept and implement these new trends.
6. The IPS may play a pivotal role in the development of a GP training programme. If GPs are trained in mental health, the psychiatric load at the secondary and tertiary levels can be minimized, thus strengthening the mental health delivery system.
7. We are proud of quite a few outstanding psychiatric educational institutions in India, which are internationally acknowledged for the high-quality research work they produce. The IPS may take a central role along with these institutions for a ‘National Data Bank’ which will update the diverse mental health data of India.

## OTHER OBSERVATIONS

### Dangerous psychopharmacological trends

The rapid discovery of different classes of psychopharmacological agents has created a trend for irrational prescriptions. The psychological management of many mild to moderate mental symptoms escape the attention of professionals because of these readily available therapeutic agents. This is a dangerous trend which alienates the mental health specialist from understanding the psychodynamic perspective of the human being. It is a global issue and, as practised in Royal College of the UK or APA in the USA, the IPS may negotiate with the University to incorporate psychotherapeutic teaching and training as a part of the psychiatric residential programme in their curriculum.

### Human resource utilization

- Lack of mental health manpower is an obstacle to the development of a comprehensive psychiatric service in the community. In spite of our best efforts, the ratio between psychiatrists and the patient population is not likely to change in the near future. The healthcare system in India is unique and if local healthcare personnel could be judiciously utilized for mental health promotion and prevention, we may overcome the shortage of manpower.
- A large number of mental health professionals are NRIs. The IPS can skilfully utilize their expertise for the benefit of our country. Some need-based programmes can be planned at the state or national level for manpower development. I would like to send messages to those NRI psychiatrists holding nodal posts to open up opportunities for short-term advanced training programmes for the budding psychiatric talent of our country.

### Brain drain

This is a problem for any developing country, including India. As a professional body, the IPS is not in a position to form any opinion. In a democratic country is it feasible to put restrictions on anybody's movement for a noble cause? My appeal to those who are yet to settle abroad permanently and come to India periodically is that they should keep some days free to conduct training programmes. In India, there are institutions and medical colleges where there is a dearth of qualified personnel. The IPS can come forward to utilize their services during their short stay in India. I would request the zonal/state officers of the IPS to keep in touch with visiting NRI psychiatrists and arrange for suitable programmes.

### Spirituality and religion

Spirituality and religion are topics of wide discussion and interest in a country with a rich heritage of religious diversity and spiritual philosophy. The IPS may develop a taskforce to frame guidelines for the effective utilization of religious or spiritual beliefs in mental health healing. This model may serve as an indigenous therapeutic approach for our multiracial, multi-cultural country.

### Quality assurance

Though quality assurance has been a necessity both for psychiatric education and mental hospitals in India, it is showing a steady downfall. Some horrible cases of transgression of human rights in some mental hospitals is a matter of deep concern. I presume the IPS can form a task force which can discharge ethical responsibilities in India.

### Attempted suicide

Deliberate self-harm and suicide is a great public health challenge. I think it is time for the IPS to urge the government to formulate a national suicide prevention programme in India.

#### Attempting suicide is a ‘crime’

On 21 March 1996, a five-judge Constitution Bench of the Supreme Court overruled the earlier verdict of 1994, where the Court had declared Sec. 309 of the IPC as unconstitutional. This judgment has created a lot of confusion and dissatisfaction among medical and mental health professionals. This concern was voiced by my predecessor at various forums. I am of the same view, as it does not reflect the present state of mind or frustrations of the person which forced him/her to take such a violent step. Parental neglect, frustrated love affairs, socioeconomic conditions, maladjustment and misunderstandings in the family, ego problems and many other factors may lead to suicide. We cannot be silent spectators but must discuss and detect ways and means to tackle these problems as life is for living and not for being cut short. I feel the IPS should spread their messages without getting frustrated and make the judiciary understand the legitimacy of their claims.

### Stigma

Stigma is a burning problem for mental health professionals. Unless this problem is tackled properly our goal cannot be attained. I am very happy to note that the IPS has taken it seriously and chalked out action plans accordingly. My appeal to all mental health professionals is to gear up for a mass movement to unveil the real truth about mental illness and to save patients and their families from alienation and apathy.

### Media, mental illness and mental health professionals

More often than not, mental patients are depicted in a sarcastic manner. Of late, the IPS has taken it seriously and some of our members have dedicated their services to improve this prevailing attitude. I feel that the media has a great role to play, not only in eradicating the stigma attached to mental illness, but also towards a holistic improvement of mental health. The media together with the IPS will definitely help to take one step forward in invoking a new mental awareness platform and ensure betterment in the quality of life of patients with mental illness.

### Subspecialties

The rapid development of the science of mental health has necessitated that at present, psychiatry should be divided into many subdisciplines such as child psychiatry, adolescent psychiatry, adult psychiatry and geriatric psychiatry. Here, I wish to focus on geriatric psychiatry.

## GERIATRIC PSYCHIATRY IN THE INDIAN CONTEXT

I would like to concentrate on the magnitude of geriatric problems, some psychosocial aspects, common psychogeriatric disorders and their management with particular reference to the Indian context. The increase in the elderly population may pose tremendous problems in the days to come if proper measures are not taken.

The term ‘old age’ refers to the last period of human life, above 65 years. Late adulthood usually begins at the age of 60 years. Though middle and late adulthood may not be clearly demarcated by any physical or intellectual transformation, the gerontological approach to studying the aged and the ageing process classifies elderly people into two categories: (i) the young old, who constitute the majority of older people irrespective of their actual age and are vital, vigorous and active; (ii) the old, who are frail and infirm and form the majority.^3^ Some developmentalists distinguish between the young old (65–75 years) and the old old (75 years and above).^4^

Some other investigators distinguished the oldest old (85 years) and older from younger old.^5^ As per the WHO guidelines people 60–74 years of age are called elderly and those between 75 and 85+ years of age as old. The oldest old are more likely to be female and they have a higher rate of morbidity than their younger counterparts. This differentiation of late adulthood into sub-periods of development is heterogeneous. Even the oldest old are a heterogeneous, diversified group.^6^

Many of the physical changes that come with age include greying of the hair which also becomes sparse, drying and wrinkling of the skin, receding gums, loss of teeth leading to a shift in facial configuration. Strength and agility reduce to a considerable extent and the sensory capacity and cognition decline.

Cognitive changes in old age can be described as a ‘mixed bag’.^7^ Some intellectual skills may remain more or less constant year after year. A key developmental task, as Eriksson puts it, is the achievement of ego integrity, i.e. the capacity to reflect positively on a life well lived.^8^ Those who achieve a sense of wholeness and integrity may also develop the kind of rich, informed and emotionally balanced perspective of reality which may be called wisdom. Without this integrity the older person feels a growing sense of despair that can be revealed in various ways—perpetual irritability and disgust or a nagging fear of death. At the core there is a sense of an unfulfilled and incomplete life.

Retirement is a traditional marker of old age in many cultures. For many people, retirement signifies the loss of a familiar routine, loss of valued social interactions, and loss of income. For others, however, retirement is a transition that offers new opportunity and freedom. People who find their jobs unrewarding are likely to welcome retirement. Some major developmental tasks of old age are adjusting to the decreasing physical strength and coming to terms with the concept of death.

Eriksson's theory focuses on coming to terms with mortality and developing ego integrity.^8^ Jung stressed a change in orientation, as people shifted from extroversion to introversion.^9^ This view was echoed in the disengagement theory, which saw withdrawal from the world as the most satisfactory adjustment to ageing. The activation theory took the opposite approach by emphasizing continued involvement in society leading to a satisfactory old age.

### Old age: The Indian perspective

A significant proportion of the population in India is old. This population will go on increasing due to improved medical facilities and a reduced child mortality rate. India is a developing country with enormous resources. For the past 50 years there has been a distinct change in the Indian family system. Joint families are turning into nuclear families.

Most of the nuclear families in our society are actually extended nuclear families with one or more members related to the spouse or parents and grandparents living in the family. The status of the elderly has also changed in the present family system. Earlier, aged members were regarded as having supreme power and their experience and wisdom were utilized to solve important family issues. However, younger members of the family are the product of a changed social system. This conflict in the value pattern makes elderly people, particularly those who are retired from service or other occupations, mentally isolated from the family.

The feeling of loneliness along with the natural age-related decline in physical and physiological functioning make them prone to psychological disturbances. In some cases elderly members of relatively rich families or aged persons who have nobody to look after take shelter in ‘old age homes’. The elderly live in these homes merely in terms of existence to complete the last phase of their lives.

Aged people, even with their declining potential, can effectively contribute to the family and society in general according to their capacity. These old persons are a great human resource and not utilizing them properly may be a national waste.

Currently, government is providing certain facilities for ‘senior citizens’. But is this enough? We should pay our respects to them by providing opportunities so that they can come forward and help build the nation with their experience and wisdom.

India is presently the second-largest country in the world. The absolute numbers will increase from 7.6 million in 2001 to 137 million by 2021. The percentage growth in the elderly population is almost double the rate of increase in the general population. This means that the burden of the older population will have to be borne by the younger, adult working group.^10^ Major issues of the elderly include their dependence in both rural (50.8%) and urban areas (57.3%). Lower rates of dependence in the rural areas show that rural families are supportive of their elderly. Moreover, elderly rural males continue to work as long as their health permits. Most of them are either day labourers or cultivators. Life expectancy has increased enormously from 42 years in 1947 to 65 years today. But the sad part is that geriatric care continues to be as neglected as ever. There is no progress in the management of common geriatric illnesses and much less so for geriatric psychiatric illnesses.

### Common psychiatric disorders in the elderly population

In the years to come, medical practitioners and psychiatrists will have to face the complicated problems of the elderly. The presentation of their psychiatric illnesses differ from that of adult patients with the same illnesses. Clinicians should have good knowledge to avoid undue problems. The presentation of some physical illnesses of the elderly are also different. In this group, physical illness often presents with psychological symptoms and vice versa. Some common mistakes during diagnosis are unnoticed medical conditions such as dementia, delirium and depression.

The best way to understand a geriatric illness is to take a detailed history. The history should focus on the changes in functional performance of the activities of daily life. The drugs consumed should be documented. Habitual intake of tea, tobacco or beverages is common. Abstinence from benzodiazepines is to be kept in mind as abstinence symptoms are much more among the elderly with chronic drug abuse. Moreover, non-prescription drugs produce significant psychiatric symptoms.^11^

After taking the history, proper physical examination of higher functioning is absolutely essential. All sensory systems should be checked as even subtle diminution in any perception particularly hearing and vision may create problems. General mental status can be assessed by the Mini Mental State Examination (MMSE).^12^ Simple laboratory tests sometimes help to clinch the diagnosis; electrolyte imbalance, anaemia, malnutrition, metabolic abnormalities, and urinary retention can precipitate or exacerbate psychiatric illness. Sometimes it is observed that even therapeutic doses of medicines produce toxic effects because of malnutrition and a low protein level/low renal clearance. Dose adjustment may be done by the formula developed by Coderojt and Goult.^13^

#### Management of geriatric illness and guidelines

The following guidelines must be kept in mind while managing elderly people.

1. Multimodal approach
2. Psychosocial intervention
3. Recent trends in the treatment of depression have taken a turn towards safer drugs (drugs working through one neurotransmitter) than older drugs (working through more than one neurotransmitter); these newer drugs are to be preferred.
4. The risk of suicide increases with a delay in the initiation of treatment.
5. Co-morbidity should be treated adequately.
6. Delay of treatment of acute infection or other illness may further worsen the mental functioning of patients with dementia.
7. All organs of elderly people are in a compromised state.
8. Pharmacodynamics and pharmacokinetics are altered with age.
9. The benzodiazepine group of drugs cause more harm than benefit.
10. The dictum of medication is to start low and go slow, and never to go high.
11. Early and sudden withdrawal of the medicines may be harmful.

#### Future recommendations for better living of the geriatric population

1. India is a vast country with different regions having different subcultures. Gerontological studies with a multi-disciplinary approach should be taken up for each of the subcultures.
2. Municipal bodies and *panchayats* in urban and rural areas, respectively, need to be sensitized to the issues of the aged so that they can be engaged in unskilled and semi-skilled occupations.
3. Governmental and non-governmental organizations have to arrange awareness programmes so that the public in general pay respect and give emotional support to the elderly. The mass media should be used to create awareness among the public.
4. Preventive measures should be taken by educating people once they attain early adulthood, so that when they approach old age there will be less chances of having geriatric problems. At this point we may take a leaf out of the *Upanishads* formulated in ancient India by enlightened religious leaders to be followed by human living. These are *Brahmacharya, Grahastha, Vanaprastha* and *Sanyas*.

Some common features of all the stages of life are non-violence, truthfulness, purity and forgiveness. External happiness can be achieved if these laws which were enunciated by our most respectful sages are observed and adhered to.

The four stages may be redefined in the context of the present social condition and lifestyle. The purpose should be to develop a sense of preparedness among adolescents. Perhaps this will help them to approach life stage by stage and minimize distress, especially during the last stage.

For the past few years the IPS has been maintaining a chord of harmony with other professional bodies of the world such as the WPA, APA, BPA, etc. This relationship has opened up newer avenues to exchange needs, expressions, views and thoughts with others. In the recent past, it has been observed that some dedicated and dignified members of the IPS have developed a liaison with other bodies abroad for the propagation and promotion of mental health.

For the betterment and development of mental health in India the IPS has never fallen behind. It has always taken prudent steps when required. It has always been a pillar of support for the poorest of the poor and also for the disadvantaged populations of Indian society.

In my opinion it is of paramount importance to maintain our integrity and identity, which is a matter of pride for us. We must also give importance to our own interests rather than just establishing relationships with other professional bodies abroad. Our mission is always to reach the neglected population of India, to invite them and provide them warmth, affection and directives under the umbrella of the IPS.

To narrate in the words of Gurudev Rabindranath Tagore—

Hethay arjo hetha anarjo hethay drabir chin
Shak hoon dal pathan mogol ek dehe holo leen
Paschime aji khuliyache dar setha hote sobe ani uphar
Dibe aur nibe milabe milibe, jabena fire
Eai bharater mahamanaber sagar tire

Hetha ekdin biram bihin mahaonkar dhoni
Hriday tantre eker mantre uthechilo rono roni
Tapasya bale eker anale bohure ahuti diya
Bived bhulilo jagay tulilo ekti birat hiya
Sey sadhanar, se aradhanar
Jnagya shalas khola aji dwar
Hethay sabare hobe milibare anata shire
Eai bharater mahamanaber sagar tire
—Bharat Teertha

(This is part of a verse ‘Bharat Teertha’ from the book *Gitanjali*—one that got him the Nobel Prize. ‘Bharat Teertha’ is basically an ode to the secular spirit of India, ‘unity in diversity’.)

Aryan and non-Aryan came
Chinese, Dravidian,
Seythian, Hun, Moghul, Pathan,
In body blent as one:

And now the West unfold its doors,
The World bears bounty from its store -
Give and receive, merge and be merged:
None will excluded be
From India's Ocean-Shore of great humanity.

Here once arose without a pause
The mighty sound of ‘Om’ -
The heart-strings resonated
With the anthem of the One.

The many being sacrificed
To the One's holy fire,
Division lost, one great soul rose
By contemplation's power

Today that sacrificial hall
of worship and essay of soul
Is open: all must mingle there,
Bound in humility
On India's Ocean-Shore of great humanity.^14^
